# Assessment of Drug-Drug Interaction in Ayder Comprehensive Specialized Hospital, Mekelle, Northern Ethiopia: A Retrospective Study

**DOI:** 10.1155/2017/9792363

**Published:** 2017-11-08

**Authors:** Zeru Gebretsadik, Micheale Gebrehans, Desalegn Getnet, Desye Gebrie, Tsgab Alema, Yared Belete Belay

**Affiliations:** ^1^Mekelle University, School of Pharmacy, Pharmacoepidemiology and Social Pharmacy Courses and Research Team, Mekelle, Ethiopia; ^2^Department of Pharmacy, Pharmacology and Toxicology Courses and Research Team, Adigrat University, Adigrat, Ethiopia

## Abstract

**Introduction:**

Adverse drug interaction is a major cause of morbidity and mortality. Its occurrence is influenced by a multitude of factors. The influences of drug-drug interactions (DDIs) can be minimized through creation of awareness to health care professionals.

**Objective:**

The objective of this study was to assess DDIs in Ayder Comprehensive Specialized Hospital (ACSH).

**Methodology:**

A retrospective study design was employed on patient prescriptions available in the outpatient department of pharmacy and filled from September 2016 to February 2017 in ACSH.

**Result:**

From the 600 prescription records assessed, the average number of drugs on single prescription was 2.73. Regarding the interaction observed 34 (9.63%) prescriptions with major drug-drug interaction, 210 (59.5%) moderate, 87 (24.65%) minor, and 22 (6.22%) unknown were identified. Age category showed significant association to affect the occurrence of DDIs and polypharmacy had statistically significant association with DDIs in bivariate analysis which was lost in adjusted OR.

**Conclusion:**

From the current study it can be concluded that nearly half of the prescription ordered in ACSH contained DDIs and from the prescription with interacting medications majority of them had moderate DDIs.

## 1. Introduction

Drug-drug interactions (DDIs) are defined as two or more drugs interacting to the extent which could alter the effectiveness or toxic effect of drugs [1]. Drug interactions can occur in several different ways basically, at pharmacokinetic, pharmacodynamics, and pharmaceutical interactions [2]. However, pharmacokinetic interactions have been the main focus in the US and Europe DDI guidelines [3]. Pharmacokinetic interaction occurs when one drug affects another drug's absorption, distribution, metabolism, and elimination when they are given concomitantly [2, 3]. Pharmaceutical interaction occurs when chemically incompatible drugs are mixed outside of the body, as, for example, phenobarbital and opioid analgesics mixing in the same syringe which will result in inactivation of one or both drugs [4, 5]. Studies have been conducted on the prevalence of potential drug-drug interactions in various clinical settings [6, 7]; however the burden of interactions has not been assessed in our set-up with an effort that addresses all wards.

However, the average number of drugs used by each patient has been increasing overtime and this increases the risk of getting DDIs. One study have shown that the prevalence of DDIs in Patients who are 55 years or older in the Netherlands increased from 10.5% to 19.2% between the years 1992 and 2005 [8, 9]. Despite the overwhelming effect of drug interactions and increased prevalence of these deleterious health outcomes attributed to drug interactions, there is a limited consensus list of drug-drug and drug-disease interactions [10] and yet there is low knowledge on characteristics of patients who encountered drug-drug interaction, as well as potential determinants of events [11]. In Ayder Comprehensive Specialized Hospital, a large number of clients receive combination of drugs. Patients with high drug interaction risks such as patients with HIV, hepatic, and renal diseases and/or elder patients are getting health care services [12]. So assessing the level of drug interaction in such health facilities' could have tremendous importance.

Worldwide reports showed that drug interactions cause around 21% of adverse drug event-related hospital admissions. A study conducted in Australia indicated that nearly 100,000 hospital admissions were associated with adverse drug events (ADEs), representing 1.3% of all hospital admissions. Another retrospective prevalence study in Australia also indicated that, out of the 287,074 subjects enrolled in the study, potentially hazardous interacting drug pairs were dispensed to 1.5% of the cases and similarly study from Swiss depicted 1.11 major and moderate DDIs were identified per patient; furthermore, this study realized that 47% of all major and moderate DDIs at hospital discharge were created during hospitalization [13–15].

Elderly patients are at higher risk of potential drug interactions and occurrence of potential drug interactions ranges from 3 to 69%, depending on the specific area and population. This increased prevalence was found to be related to presence of multiple chronic illnesses in the elderly patients and their tendency to receive a combination of drugs. Age related physiologic (a decrease in renal and hepatic functions) and drug disposition change might aggravate their clinical conditions [16]. In addition these population segments have a tendency to use alternative complementary medicines such as herbal medicines which can affect drug metabolism [13]. Retrospective medical record review in New York in 2006 disclosed that HIV/AIDS patients who receive antiretroviral therapy commonly encounter clinically significant drug interactions. In this study, out of 153 patients, 63 (41%) had clinically significant drug interactions which required dosage adjustment [16]. Another study also claimed that clinically significant drug interactions are highly prevalent among HIV-infected patients receiving antiretroviral therapy. Familiarization with the most common CSDIs provides clinicians with valuable information to the recognition of risk factors for CSDIs and help them identify patients with the greatest need for drug interaction evaluation during prescribing antiretroviral therapy. Therefore, to get improved clinical outcomes, clinicians should have knowledge of the risk factors for CSDIs to recognize and manage CSDIs [17].

A cross-sectional study conducted in Ayder Referral hospital indicated that drug utilization pattern in the setting was not according to the WHO prescribing criteria, and there is increasing polypharmacy and overprescription of antibiotics tendency was found increased. In this study 1003 drug products were analyzed for potential DDIs out of 384 prescriptions. Out of 305 prescriptions which contain two or more drugs, clinically significant drug-drug interactions were detected in 109 (31%) of prescriptions [7].

The aim of this research was to assess the pattern and predictor of drug-drug interaction in Ayder Comprehensive Specialized Hospital.

## 2. Methods

### 2.1. Study Area

ACSH is located in Tigray, Ethiopia. It is one of the specialized teaching hospitals in Tigray regional state. ACSH commenced its referral and nonreferral services in 2008 to more than 8 million populations in its catchment areas of Tigray, Afar, and Northeastern parts of the Amhara Regional States. It provides a broad range of medical services to both in- and outpatients of all age groups. It has a total capacity of about 500 inpatient beds in four major departments and other specialty units; the ACSH is also used as a teaching hospital for the College of Health Sciences, Mekelle University. ACSH has four pharmacy units, namely, OPD Pharmacy, Inpatient Pharmacy, ART Pharmacy, and Emergency Pharmacy.

### 2.2. Study Design and Period

An institution based retrospective cross-sectional study was conducted from September 2016 to February 2017

### 2.3. Source and Study Population

All prescriptions received by patients who came for medical service in ACSH were the source population and all prescriptions dispensed to patients in ACSH from OPD pharmacy through period of September, 2016, to February, 2017, were the study population.

#### 2.3.1. Inclusion Criteria

Prescriptions of patients attending the hospital from September, 2016, to February, 2017, were included.

#### 2.3.2. Exclusion Criteria

Patients prescribed only one prescription and prescriptions with unclear data were excluded.

### 2.4. Study Variables

#### 2.4.1. Dependent Variable

The dependent variable is DDIs.

#### 2.4.2. Independent Variable

The independent variables are sociodemographic characteristics of the patients and polypharmacy.

### 2.5. Sample Size Determination and Sampling Technique

Sample size is determined as follows:

(1) $$\begin{array}{c} n=\frac{Z^{2}p\left(1-p\right)}{d2}, \end{array}$$

where *n* is sample size.

p is proportion taken as 0.5, because there is no study showing the proportion of the population exposed to DDIs in ACSH. *d* is margin of sampling error tolerated, 0.05. *Z* is the standard normal value at confidence interval of 95% = 1.96. Therefore, *n* = (1.96)^2^0.5(1 − 0.5)/(0.05)^2^ = 384. 5% for nonresponse rate in the total sample size is 403.

But the smallest sample size recommended by WHO for drug utilization study is 600 and this sample size was considered as a sample of study in this work. First the six-month prescriptions were selected from September 2016 to February 2017 and all the prescriptions were assembled in their respective months to form six strata and from each stratum 100 prescriptions which fulfilled the selection criteria were selected by lottery method.

### 2.6. Data Quality Control

A pretest was carried out in 30 prescription papers from the prescriptions other than those of the study period, in order to check the feasibility of data collection checklists. The data collection process was controlled by the principal investigator. The collected data checked out for the completeness, accuracy, and clarity.

### 2.7. Data Processing and Analysis

**Figure 1:**
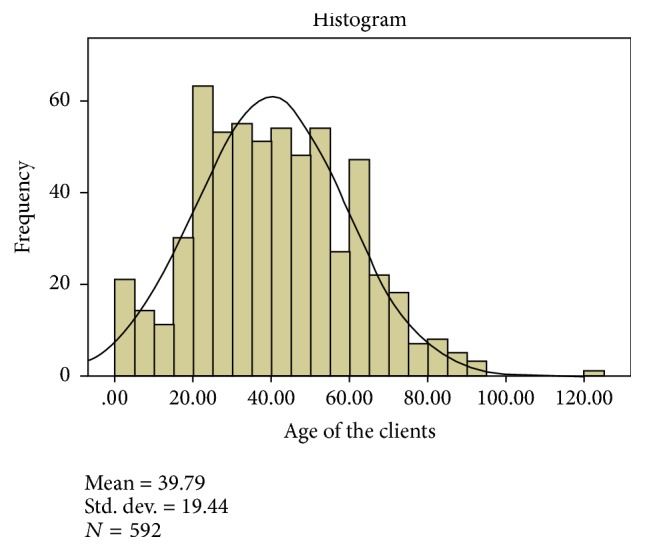
Age distribution of the clients from record of the prescription, Mekelle, ACSH May 2017.

**Figure 2:**
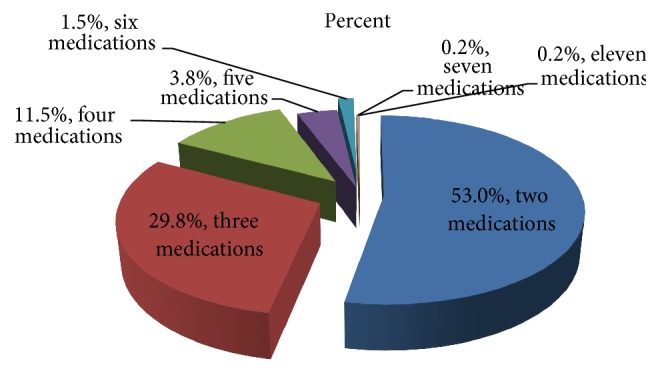
Frequency distribution of number of drugs prescribed in single prescription paper, Mekelle, ACSH May 2017.

**Figure 3:**
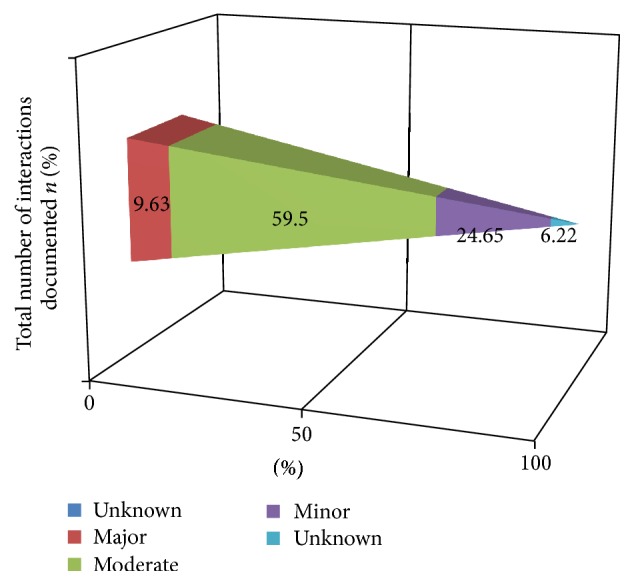
Frequency distribution of number of DDIs from the total prescription paper, Mekelle, ACSH May 2017.

Data analysis was done by using SPSS version 23 after data is checked for errors and coded to numerical values. The data was summarized with descriptive statistics using Tables 1, 2, and 3 and Figures 1, 2, and 3 for displaying results. To see the effect of independent variables on the outcome variable, bivariate analysis was conducted and variables which showed *P* value less than 0.2 in bivariate analysis were considered for multivariate analysis.

### 2.8. Ethical Considerations

The ethical clearance for this study was obtained from Ethical Review Committee of School of Pharmacy, Mekelle University, and permission to conduct the study was granted from hospital pharmacy head and medical director of ACSH. After permission and approval are secured data was collected from patient prescriptions. In doing so, personal identifiers of prescriptions were not used and confidentiality of information was maintained to use data for only the intended purpose.

### 2.9. Limitation of the Study

This retrospective study reviewed prescription papers, so the data quality and completeness could be the possible limitation of the study; now and then all medications that the patient took may not be listed on the perception paper and there might be discrepancy in actual number and type of medication that the patient received and obtained from the prescription records. The study analyzed drug interaction by using the online software drugs.com interaction checker and the software only shows the type of interaction as major, minor, and moderate but not the strength of evidence/documentation and other clinically relevant evidences like onset of interaction and nature of interaction as pharmacokinetic or pharmacodynamics level.

### 2.10. Sociodemographic and Related Profile

From the selected prescription more than half (52.2%) of them were orders for female clients and only 5.67 percent of prescriptions were prescriptions with polypharmacy. From the prescriptions ordered 45.9% of them were drugs with different level of interaction.

The age distribution of the study participants was normally distributed with the mean age 39.79 and standard deviation of ±19.44.

#### 2.10.1. Descriptive Statistics of DDIs

From the 600 prescription records assessed, average number of drugs on single prescription was 2.73.

Regarding the interaction observed 34 prescriptions with major DDIs, 210 moderate, 87 minor, and 22 unknown were identified. The medication interactions were categorized as major, moderate, minor, and unknown according to the findings of the online drug interactions checker called drugs.com. The unknown drug-drug interactions are categorized in such a group because, some of the medications were not available in the database of the interactions checker and such prescriptions were categorized as prescriptions which contain unknown drug-drug interactions.

From the total prescriptions with drug interaction no prescriptions contained more than single major drug interaction while 20.47% of the prescriptions with moderate interactions contained two moderate DDIs. From those prescriptions with moderate DDIs, 4.76% of the prescriptions were recorded with more than four moderate DDIs on single prescription paper.

#### 2.10.2. Predictors of DDIs

The impact of independent variables like gender and age of the clients from sociodemographic profiles and number of the drugs on the occurrence of DDIs was analyzed by multinomial regression. Age category showed significant association to affect the occurrence of DDIs. Polypharmacy had statistically significant association with DDIs in bivariate analysis which was lost in adjusted OR.

## 3. Discussion

This study aimed to assess DDIs in ACSH, Mekelle, Northern Ethiopia. From the current study, more than half (52.2%) of the prescriptions were ordered for female clients; the gender distribution of the prescription pattern in this study showed slightly higher female proportion, but the study from Dessie Referral Hospital depicted much higher proportion of prescription with female clients; furthermore, this could be expected difference as it was supported by the study from Princeton University which revealed that hospitalization, mortality, and worsened health condition were favorable toward female gender than males [18, 19]. From the current study only 5.67 percent of prescriptions were a prescription with polypharmacy (single prescription with more than five medications). The finding of this study is lower than Bhopal district in India which depicted 8.73% of prescriptions with polypharmacy [20]. Polypharmacy has a lot of consequences like increased health care cost, adverse drug reactions, nonadherence of the clients, functional decline in elderly patients, cognitive impairment, decline in nutritional status, and precipitation of morbidity and mortality conditions [21].

This retrospective study assessed a total of 600 prescriptions and, from this, 275 (45.9) of the medication orders had drug interaction from major to minor. The prescription/sample/size of the study was determined by standard of WHO which settled for drug utilization studies in health facilities and this is the minimum sample size according to the standard [22]. This study depicted the average number of drugs prescribed on single prescription paper to be 2.73 which is lower than the result reported from Nepal which revealed average number of drugs per prescription as 3.76. This difference might be the result of the difference in disease characteristics of the participants. In the current study the prescriptions were sampled without considering types of the disease and the study of Nepal involved only DM patients [23]. From the current study, the ratio of prescription with drug interaction is lower in comparison to study from Iran that published that 91.43% clients have shown potential DDIs; in fact the study of Iran was conducted in intensive care unit [24]. Another study from India depicted 69.3% of potential drug interaction from the prescribed medications in tertiary care hospitals, yet this finding is significantly greater than the current study [25]. However study which assessed prescription from community and hospital pharmacy in Pakistan reported that 40% of the prescribed orders had interacting drugs within their order; this is lower in comparison to the finding of the current study. Except the sample size the characteristics of the prescription and the methodology of the two studies are similar; the difference might be the result of difference in institutions involved in the study: the study of Pakistan involved community pharmacies and in case of the current study they were not considered [26].

This study includes prescription with two and more numbers of medications and from the total number of prescriptions assessed 53% of the prescriptions were orders of two medications followed by a prescription which includes three medications and it accounts for 29.8%; this is comparable with the result reported from India which showed similar decline direction in number of prescriptions which had higher number of medications per single prescription even though the rate of decline is higher in the current study. The prior study conducted in India reported that, from the prescriptions involved in study, 160 prescribed 2 drugs, followed by 152 prescriptions which had 3 drugs [27]. In this study severity of drug interactions was assessed by using drugs.com online service. From the software drugs combination with no DDIs and major, moderate, and minor drug-drug interactions were identified and those prescriptions with list of medications which are not in the database of drugs.com were considered as a combination of medications with unknown DDIs.

From the analysis 34 (9.3%) prescriptions with major DDIs, 210 (59.5%) moderate DDIs, and 87 (24.65%) minor DDIs were identified. In comparison to report from India this study depicted lower rate of major and moderate DDIs and higher minor drug interactions; the result of the Indian study published indicated 18.94%, 68.72%, and 12.33% major, moderate, and minor drug interactions, respectively, so from this comparison it might be possible to appreciate the prescription patterns of ACSH as one of the tertiary care hospitals in least developed countries [25].

From this study difference in age category showed significant association to affect the occurrence of DDIs and those with age category less than 18 showed less risk of having DDIs (AOR = 0.403 [0.209–0.778]) and polypharmacy had statistically significant association with DDIs in bivariate analysis (COR = 0.744 [95% CI, 0.630–0.879]) which was lost in adjusted OR. The result of polypharmacy was not expected in this manner because of the high likelihood of association between polypharmacy and occurrence of DDIs [28]. This result might be due to the lower number of sample size and particularly small number of prescriptions with polypharmacy in comparison to large number of prescriptions without polypharmacy. Another study from India also showed increased number of DDIs with the higher number of drugs per prescription, even though the association of polypharmacy with DDIs lost in adjusted odds ratio was observed in bivariate analysis, thus making the finding of this study slightly similar to that of the Indian study [25].

## 4. Conclusion

From the current study it can be concluded that nearly half of the prescriptions ordered in ACSH contained DDIs; from the prescriptions with interacting medications majority of them had moderate DDIs. The study also depicted that difference in age category had association with the occurrences of DDIs and clients with age category below 18 showed less likelihood of having medications with interaction on their prescriptions; furthermore, the study tried to reveal that polypharmacy also could have an impact on the rate of DDIs.

## 5. Recommendation

From the finding of this study it is possible to recommend that health professionals should follow guidelines and they have to use references for preventing the occurrences of unnecessary DDIs; health amenities and health bureaus should avail different guidelines for prescribers and dispensers for referencing purposes; ministry of health and regulatory authority should prepare updated guidelines which provide evidence based prescribing patterns. All stakeholders in health care arena should contribute their shares for installing different software like Micromedex, drugs.com, and others for quick references. Despite this dispensaries and other health professionals should counsel their clients not to take a lot of pills without the knowledge of health professionals in order to prevent PDDIs.

## Figures and Tables

**Table 1:** Sociodemographic and related profile of the clients from record of the prescription, Mekelle, ACSH May 2017.

Variable	*n*	Frequency *n* (%)
Gender		
Male	596	291 (48.8)
Female		305 (52.2)

Age group (years)		
<18	592	56 (9.5)
18–49		344 (58.1)
>49		192 (32.4)

Polypharmacy		
Yes	600	34 (5.67)
No		566 (94.33)

Drug interaction		
Yes	599	275 (45.9)
No		324 (54.1)

**Table 2:** Pattern of DDIs from the prescription records, Mekelle, ACSH May 2017.

Type of interaction	Number of interactions that occurred on individual prescription paper	Total number of interactions documented *n* (%)
Major	1	34 (100)

Moderate	1	134 (63.8)
	2	43 (20.47)
	3	23 (10.95)
	≥4	10 (4.76)

Minor	1	65 (74,71)
	2	18 (20.69)
	≥3	4 (4.6)

Unknown	1	21 (95.5)
	>1	1 (0.5)

**Table 3:** Predictors of DDIs from the prescription records, Mekelle, ACSH May 2017.

Variable	Drug interaction occurred		Crude OR [95% CI]	Adjusted OR [95% CI]
	Yes (%)	No (%)		
Sex				
Male	141	150	1.193 [0.864–1.647]	1.123 [0.799–1.578]
Female	134	171	1	1

Age group (yrs)				
<18	16	40	0.301 [0.158–0.575]	0.403 [0.209–0.778]
18–49	148	196	0.568 [0.397–812]	0.734 [0.503–1.071]
49+	109	82	1	1

Polypharmacy				
Yes	34	0	0.744 [0.630–0.879]	0.958 [0.669–0.1.373]
No	241	324	1	1
